# ReporTree: a surveillance-oriented tool to strengthen the linkage between pathogen genetic clusters and epidemiological data

**DOI:** 10.1186/s13073-023-01196-1

**Published:** 2023-06-15

**Authors:** Verónica Mixão, Miguel Pinto, Daniel Sobral, Adriano Di Pasquale, João Paulo Gomes, Vítor Borges

**Affiliations:** 1 Genomics and Bioinformatics Unit, Department of Infectious Diseases, National Institute of Health Doutor Ricardo Jorge (INSA), Lisbon, Portugal; 2 National Reference Centre (NRC) for Whole Genome Sequencing of Microbial Pathogens: Database and Bioinformatics analysis (GENPAT), Istituto Zooprofilattico Sperimentale Dell’Abruzzo E del Molise “Giuseppe Caporale” (IZSAM), Teramo, Italy

**Keywords:** ReporTree, Genetic clustering, Genomic surveillance, Public health, Automated pipeline

## Abstract

**Background:**

Genomics-informed pathogen surveillance strengthens public health decision-making, playing an important role in infectious diseases’ prevention and control. A pivotal outcome of genomics surveillance is the identification of pathogen genetic clusters and their characterization in terms of geotemporal spread or linkage to clinical and demographic data. This task often consists of the visual exploration of (large) phylogenetic trees and associated metadata, being time-consuming and difficult to reproduce.

**Results:**

We developed ReporTree, a flexible bioinformatics pipeline that allows diving into the complexity of pathogen diversity to rapidly identify genetic clusters at any (or all) distance threshold(s) or cluster stability regions and to generate surveillance-oriented reports based on the available metadata, such as timespan, geography, or vaccination/clinical status. ReporTree is able to maintain cluster nomenclature in subsequent analyses and to generate a nomenclature code combining cluster information at different hierarchical levels, thus facilitating the active surveillance of clusters of interest. By handling several input formats and clustering methods, ReporTree is applicable to multiple pathogens, constituting a flexible resource that can be smoothly deployed in routine surveillance bioinformatics workflows with negligible computational and time costs. This is demonstrated through a comprehensive benchmarking of (i) the cg/wgMLST workflow with large datasets of four foodborne bacterial pathogens and (ii) the alignment-based SNP workflow with a large dataset of *Mycobacterium tuberculosis*. To further validate this tool, we reproduced a previous large-scale study on *Neisseria gonorrhoeae*, demonstrating how ReporTree is able to rapidly identify the main species genogroups and characterize them with key surveillance metadata, such as antibiotic resistance data. By providing examples for SARS-CoV-2 and the foodborne bacterial pathogen *Listeria monocytogenes*, we show how this tool is currently a useful asset in genomics-informed routine surveillance and outbreak detection of a wide variety of species.

**Conclusions:**

In summary, ReporTree is a pan-pathogen tool for automated and reproducible identification and characterization of genetic clusters that contributes to a sustainable and efficient public health genomics-informed pathogen surveillance. ReporTree is implemented in python 3.8 and is freely available at https://github.com/insapathogenomics/ReporTree.

**Supplementary Information:**

The online version contains supplementary material available at 10.1186/s13073-023-01196-1.

## Background

Whole-genome sequencing (WGS) is the method with the highest resolution to discriminate and classify microorganisms (either at inter- or intra-species level) based on their genetic relatedness. Therefore, the implementation of genomics-informed surveillance systems able to track the circulation of pathogens and monitor their clinical and epidemiologically relevant features is essential for infectious diseases’ prevention and control and for a more informed public health decision-making.

Several bioinformatics solutions for the analysis of WGS data are currently available, with most workflows for genetic clustering determination ending up in the same key output: a phylogenetic tree or a tree-like representation. This often corresponds to a minimum spanning tree (MST) or a dendrogram reflecting the allele distances that result from a core-genome (cg) or whole-genome (wg) multilocus sequence type (MLST) analysis (commonly used approach for bacterial pathogens [1]) or to a rooted tree reflecting the single-nucleotide polymorphism (SNP) distances that result from a multiple sequence alignment (e.g., as routinely applied for viruses [2], such as SARS-CoV-2 or monkeypox virus). Subsequently, the identification and characterization of epidemiologically/biologically relevant genetic clusters (e.g., clusters of outbreak-related strains) often consists of the visual exploration of these (large) phylogenetic trees and associated metadata, taking advantage of robust visualization tools, such as those provided by PHYLOViZ [3], GrapeTree [4], Nextstrain [5], Microreact [6], or Taxonium [7]. As such, this task can be time-consuming and difficult to reproduce.

In this context, there is a continuous scientific effort to automate the identification of clusters at specific genetic thresholds [4, 8–14] and develop dynamic cluster/lineage nomenclature systems, such as the Pango system for SARS-CoV-2 [15] or the bacteria-oriented “SNP address” of SnapperDB, the HierCC of Enterobase, the “HashID” of chewieSnake, or the INNUENDO nomenclature system [9, 10, 14, 16]. Still, the field would benefit from the development of automated and more flexible tools that can be used for a wide variety of species, not only to facilitate the detection of genetic clusters at any (or all) distance thresholds of a tree but also to automatically characterize them based on the available metadata variables of interest.

Here, we present ReporTree, an automated surveillance-oriented resource that allows diving into the complexity of pathogen diversity to rapidly identify genetic clusters at any distance thresholds between samples and further characterize them according to any relevant epidemiological indicator in a reproducible manner. ReporTree’s flexibility, reproducibility, and performance make it an innovative resource to enhance existing genomics surveillance systems, with potential benefits at multiple pathogen levels.

## Implementation

**Fig. 1:**
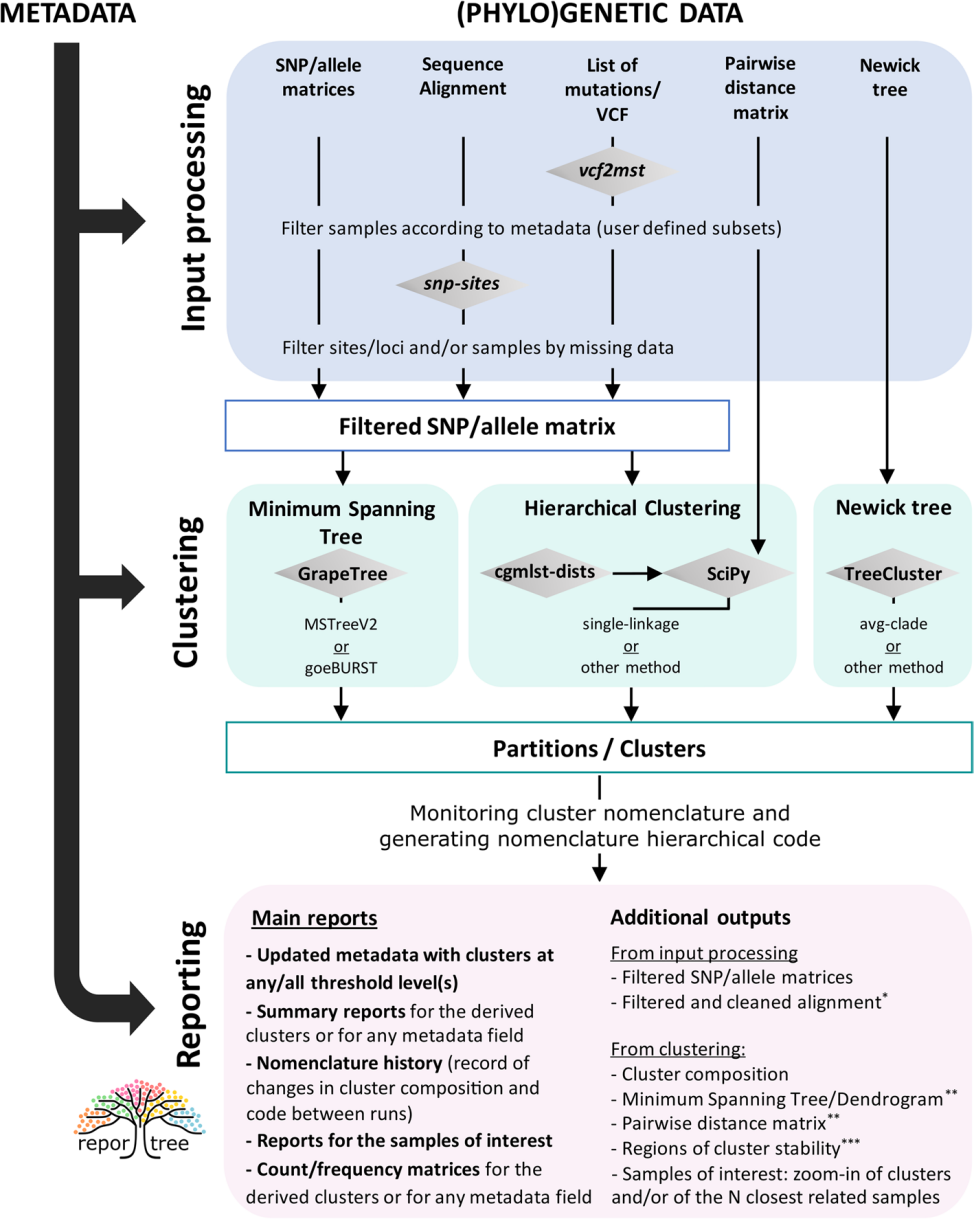
Schematic representation of the three main steps of ReporTree pipeline. Blue background highlights the alternative input types, green background highlights the alternative clustering modules, and pink background highlights the main outputs of ReporTree. Arrows indicate the alternative workflows for each input. Single asterisk, only output when a sequence alignment is provided. Double asterisks, exclusive output of MST and HC analysis. Triple asterisks, output of an optional step (comparing partitions) not represented in the figure

ReporTree is a command-line tool implemented in python 3.8 that represents a flexible solution to obtain clustering information at any sample distance thresholds (partitions) either for species that require a cg/wgMLST analysis or for those that rely on SNPs/multiple sequence alignments for tree reconstruction. As shown in Fig. 1, ReporTree pipeline can be divided into three major steps:

### Input processing

The methodology used for WGS data analysis varies from species to species. For this reason, ReporTree was carefully designed to accept multiple input formats (Table 1), being suitable for application in a wide variety of pathogens. Besides SNP/allele matrices and trees/dendrograms in Newick format, ReporTree accepts other input formats such as multiple sequence alignments, VCF files, or distance matrices. For instance, when a multiple sequence alignment is provided, ReporTree runs the script *alignment_processing.py* (also available in standalone mode) to clean the alignment according to the user's specifications and to convert it into a SNP matrix that will be used in the remaining steps. Moreover, when sample genetic variability is provided in the format of multiple VCF files or a list of mutations (variant sites), ReporTree uses vcf2mst [17] to do this format conversion. Besides the input transformation, ReporTree’s input processing step can also involve the filtration of the input files to (i) remove samples with excess of missing data (e.g., samples with less than 95% of cgMLST loci called), (ii) remove informative sites/loci from the SNP/allele matrices (e.g., wgMLST loci called in less than 98% samples), and (iii) analyze a subset of samples fulfilling the metadata parameters specified by the user (e.g., samples from a given sequence type [ST] or year) (Fig. 1). This dynamic approach allows maximizing the loci/positions shared by a subset of samples, thus contributing to an increased resolution power and, consequently, a higher confidence in the clustering analysis, aligned with a previously explored rationale [14].

**Table 1:** Summary of ReporTree input types and respective clustering options, with indication of the main outputs provided by this tool

Inputs Metadata and (phylo)genetic data	Clustering options	Main outputs
- **Multiple sequence alignment** (e.g., core SNP alignment) - **SNP/allele matrix** (e.g., derived from cg/wgMLST analysis) - List of **mutations** or **VCFs** - Pairwise **distance matrix** (only for HC)	**Minimum spanning tree** (using GrapeTree)	- **Genetic clusters** at any (or all) possible distance threshold(s) (partitions table) - **Updated metadata** table with clustering information (and nomenclature) - **Summary reports with the statistics/trends for the derived genetic clusters** - **Nomenclature history (record of changes in cluster composition and codes between runs)** - Summary reports and in-depth cluster analysis for **samples of interest** - **Count/frequency matrices for the derived genetic clusters** or for any other indicated grouping variable - **Regions of cluster stability** - **Newick tree** (when applicable)
	**Hierarchical clustering** (using several methods, such as single-linkage)	
- **Newick tree** (e.g., SNP-scaled tree or dendrogram)	**Distance between leaves and root or between tree nodes** (using TreeCluster)	

### Clustering

#### Cluster detection

Once the input files have been processed, ReporTree determines genetic clusters at all user-defined partition thresholds. When the input file corresponds to a phylogenetic tree, ReporTree runs the script *partitioning_treecluster.py* (also available in standalone mode), which takes advantage of TreeCluster [8] to automatically determine the genetic clusters using one or several of the different algorithms provided by this tool. For all the other input types (except for the distance matrix which can only be used with a hierarchical clustering (HC) approach), the user can choose between genetic clustering using a GrapeTree or a HC algorithm (Fig. 1). When a GrapeTree algorithm is requested (MSTreeV2 or goeBURST [4, 18]), ReporTree runs the script *partitioning_grapetree.py* (also available in standalone mode), which uses a modified version of this program [19], to obtain the MST and all the genetic clusters. When a HC algorithm is requested, ReporTree runs the script *partitioning_HC.py* (also available in standalone mode), which calculates pairwise Hamming distances with cgmlst-dists [20] and determines the genetic clusters using one or several of the different algorithms provided by SciPy [21]. The main output of each of these three clustering options is always a so-called partitions table with clustering information for each sample at all the distance thresholds and clustering algorithms requested by the user. Additionally, if the user provides a list of samples of interest (e.g., outbreak-related or newly sequenced samples), ReporTree can automatically apply the above-mentioned dynamic approach by additionally running a high-resolution analysis for every cluster including samples of interest and/or for the subset of the “N” most closely related samples to the samples of interest.

As a complement, ReporTree can optionally identify ranges of distance thresholds associated with cluster stability, i.e., subsequent partition thresholds in which clustering composition is similar. This kind of analysis can be useful for the user in future pathogen-specific nomenclature design. If requested by the user, ReporTree determines those “stable” regions by running a modified version of the code of the Comparing Partitions tool [22, 23]. This new version [24] takes as input the “partitions table” with clustering information at all possible thresholds and assesses several metrics (Simpson’s Index of Diversity, Adjusted Rand and Adjusted Wallace coefficient) to compare the clustering information at consecutive partitions (from “n + 1” to “n”). Based on a previously described approach [22, 25], ReporTree then uses the neighborhood Adjusted Wallace coefficient (nAWC) to ultimately determine regions of cluster stability [14, 26].

#### Cluster nomenclature system

ReporTree includes a nomenclature system that can act in two different but complementary ways, namely by (i) maintaining cluster nomenclature at any or all distance thresholds over time and (ii) providing a nomenclature code for each sample that combines clustering information at different hierarchical levels. For the first approach, the intended usage is to provide as input a “partitions table” containing the cluster names at any or all distance thresholds of a previous ReporTree run, which will then be used to (re)name the clusters (for the respective thresholds) of the current run. With this approach, clusters that do not change their composition or just acquire new samples (most expected scenario in a context of continuous surveillance) maintain their name. Moreover, if a previous cluster (e.g., cluster_1) is split into several new clusters in the new run, it will also keep the name by adding an additional suffix (e.g., cluster_1.1, cluster_1.2, etc.). New cluster names will be attributed in some situations, such as the merge of previous clusters, singletons that integrate clusters, and clusters exclusively composed by new samples. ReporTree keeps track of all these changes in cluster composition and nomenclature in a comprehensive tabular output. To increase the flexibility of the nomenclature system, ReporTree also allows the users to change the regular expression for cluster nomenclature (i.e., starting with “cluster_” or “singleton_”) by other nomenclature of interest (e.g., ECDC EpiPulse cluster ID, other official codes for outbreaks, genogroups, etc.), which will be kept afterwards.

Towards the simplification of the system, ReporTree can provide a short nomenclature code for each sample representing a combination of its clustering at different hierarchical levels, following the rationale behind “SNP address” and INNUENDO nomenclature systems [9, 14]. For example, if “150,30,7” thresholds are indicated, a combining code of cluster names at these levels will be generated by the same order: C3-C2-C1. In ReporTree, these levels are not predefined by default but instead must be indicated by the user, making it suitable for application to multiple pathogens and easily adaptable to the dataset diversity. ReporTree also opens the possibility to add an extra layer of information to this code with the inclusion of information of a given metadata variable (e.g., C3-C2-C1-CountryA, if country is added to the code).

### Summary report

The final step of ReporTree is the generation of summary reports with the *metadata_report.py* script (also available in standalone mode). Following the user’s specifications, this script can perform cluster characterization according to any relevant epidemiological indicator present in the metadata (e.g., source, vaccination status, antibiotic resistance phenotype). Similar summary reports can be generated to assess the distribution of any (and as many) user-specified variables of interest (e.g., ST distribution by year). When the time variable “date” is provided in the metadata, ReporTree automatically infers other time units (ISO week and ISO year) and metrics (e.g., cluster timespan) relevant for surveillance purposes. Moreover, ReporTree can provide count/relative frequency reports for any grouping variable, such as the relative frequency of the different (sub-)lineages/clusters circulating in the country over time. Noteworthy, ReporTree allows requesting specific reports for sample(s) of interest, as well as the application of filters in the metadata table to select subsets of samples that will be included in the analysis/report (without the need of generating a new subset metadata table). Moreover, when the “nomenclature code” was requested, summary reports of this variable are automatically provided, facilitating cluster tracking and characterization.

Besides these main reports (Fig. 1), ReporTree generates multiple parallel outputs that enable a fine exploration of intermediate data (e.g., pairwise distance matrices, filtered alignments, trees/dendrograms), while rendering standardized formats that can be easily explored through multiple compatible visualization tools. For instance, users can interactively visualize and explore the ReporTree derived clusters by uploading an updated metadata table (with cluster information) together with the original/derived Newick MST/dendrogram to interactive tools, such as auspice.us [5], Microreact [6], or GrapeTree [4]. In particular, ReporTree outputs can also be uploaded to GrapeTree-GIS [27] to get an interactive visualization of a MST together with temporal and geographical data.

ReporTree is available as a github repository [28, 29] or as a docker image [30].

## Results and discussion

### Benchmarking

#### cg/wgMLST workflow

ReporTree benchmarking for the cg/wgMLST workflow was performed in a laptop [Intel Core i5(R)] with 16 GB of RAM using four different datasets of distinct foodborne bacterial pathogens: *Listeria monocytogenes* (1874 isolates [31])*, Salmonella enterica* (1434 isolates [32])*, Escherichia coli* (1999 isolates [33]), and *Campylobacter jejuni* (3076 isolates [34]). Each of these datasets consists of a collection of genome assemblies and respective allelic profiles of isolates with public sequencing data (deposited in SRA/ENA) that were carefully selected to cover a wide genetic diversity (assessed in terms of ST or serotype, depending on the species). Details on the methodology used for the isolates’ selection and dataset curation can be found in each dataset repository [31–34]. Briefly, the genome assemblies were performed with Aquamis v1.3.9 [35] using default parameters. cg/wgMLST profiles were determined with chewBBACA v2.8.5 [36] using the 1748-loci Pasteur cgMLST schema for *L. monocytogenes* [37], the 8558-loci INNUENDO wgMLST schema for *S. enterica*, the 7601-loci INNUENDO wgMLST schema for *E. coli*, and the 2794-loci INNUENDO wgMLST schema for *C. jejuni* [14]. All these schemas were retrieved from chewie-NS [38] in May/June of 2022. As there was the need to determine a set of core loci for *S. enterica*, *E. coli*, and *C. jejuni* datasets, three sets of core loci were obtained for each species with ReporTree by setting distinct “--site-inclusion” thresholds: 0.95, 0.98, and 1.0 (i.e., only keep loci called in at least 95%, 98%, and 100% of the dataset samples). This resulted into cgMLST schemas with 3261, 3179, and 874 loci for *S. enterica*, 2826, 2704, and 465 loci for *E. coli*, and 1012, 987, and 29 loci for *C. jejuni*, at 0.95, 0.98, and 1.0 thresholds, respectively. As for each of these species, the values obtained at 0.95 and 0.98 were relatively similar, and in the range of what was previously determined for other datasets of the same species [14], the benchmarking proceeded with the loci obtained at the 0.98 threshold.

To assess the time performance of ReporTree with different dataset sizes, for each species, we generated sub-datasets of randomly selected isolates. The size of these sub-datasets varied between 200 and the maximum number of isolates of the respective dataset (in a 200-isolates step), with ten replicates being run per sub-dataset size. ReporTree was run for each replicate setting “--loci-called 0.95” (i.e., including only samples with at least 95% of loci called) and requesting summary reports at all possible partition levels obtained with (i) GrapeTree analysis (MSTreeV2 algorithm) and (ii) HC analysis (single-linkage algorithm). Additional runs were performed for each dataset using all isolates but requesting summary reports only for thresholds of “stability” regions (using nAWC, as described above) or at potential “outbreak” level (according to previously described cutoffs) [14, 39].

**Fig. 2:**
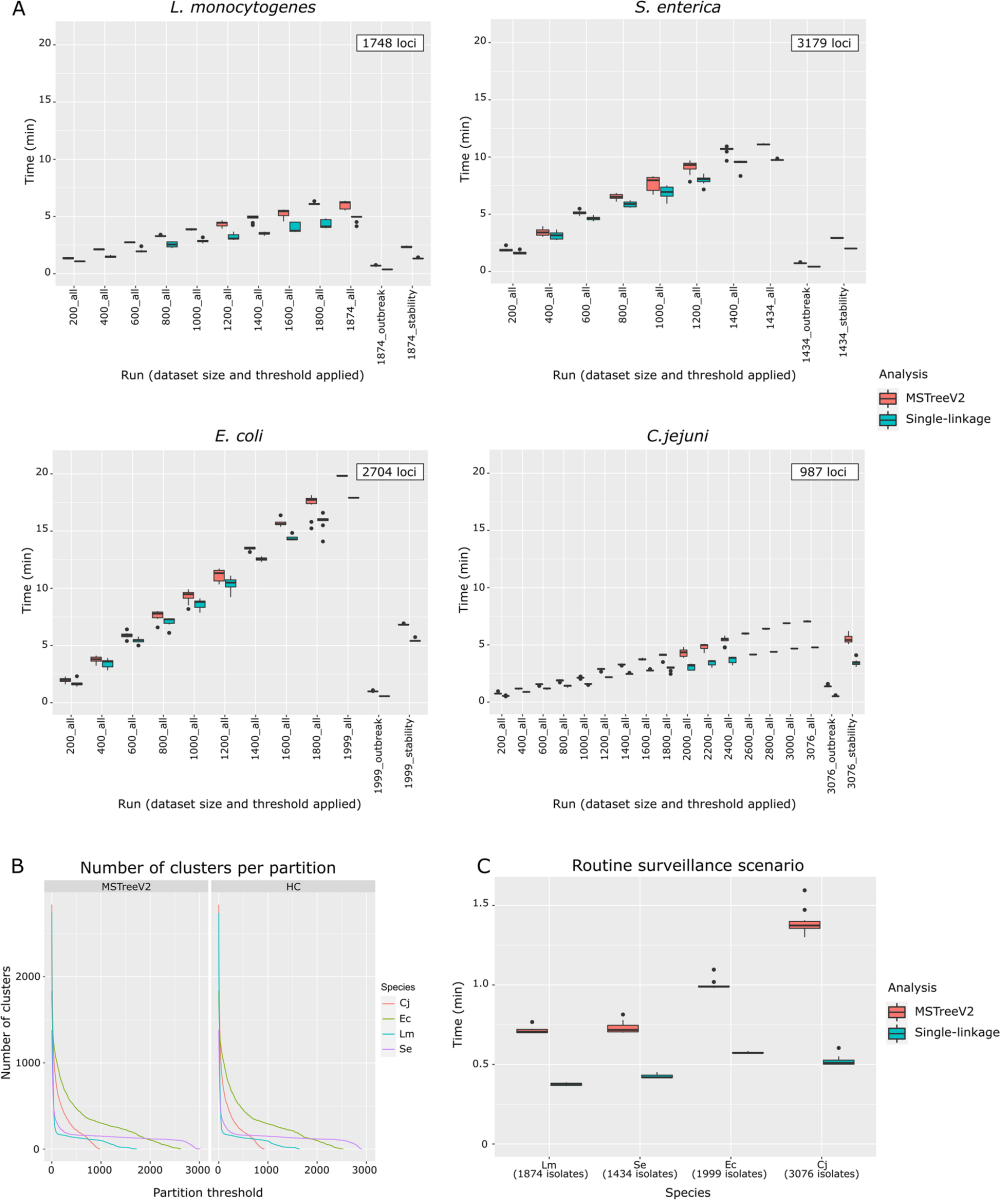
Results of ReporTree benchmarking of the cg/wgMLST workflow using datasets for four different species: *L. monocytogenes* (Lm), *S. enterica* (Se), *E. coli* (Ec), and *C. jejuni* (Cj). **A** ReporTree running times for the 10 replicates of each subset of *L. monocytogenes* (top left), *S. enterica* (top right), *E. coli* (bottom left), and *C. jejuni* (bottom right), where the flag “all” indicates subsets for which ReporTree obtained clusters at all possible thresholds, the flag “outbreak” indicates subsets for which ReporTree obtained clusters at potential outbreak level (7 allelic differences for *L. monocytogenes*, 14 (0.43%) for *S. enterica*, 9 (0.34%) for *E. coli*, and 6 for (0.59%) for *C. jejuni* [14, 38]), and the flag “stability” indicates subsets for which ReporTree obtained clusters at all possible thresholds but only generated reports for those corresponding to stability regions. **B** Number of clusters generated at all possible distance thresholds for each dataset. **C** Comparison of running times when ReporTree obtained clusters at potential outbreak level

As shown in Fig. 2A, for each dataset, ReporTree running time increases linearly with the number of samples, with GrapeTree MSTreeV2 algorithm taking slightly more time than HC single-linkage. Moreover, as expected, a higher number of loci also led to an increased running time. An interesting observation regards the comparison of *S. enterica* and *E. coli* results. Indeed, although the *E. coli* analysis involved a lower number of loci and only a slightly higher number of samples, its running times were higher than those observed for *S. enterica.* This is related to a consistent higher number of clusters in *E. coli* than *S. enterica* dataset at the same threshold (Fig. 2B), showing that, as expected, the dataset diversity also impacts ReporTree running times. Overall, using the whole dataset, ReporTree identified and characterized clusters determined at all possible distance thresholds with single-linkage and MSTreeV2 in around 5 and 6 min for *L. monocytogenes*, 10 and 11 min for *S. enterica*, 18 and 20 min for *E. coli*, and 5 and 7 min for *C. jejuni*, respectively. Nevertheless, in a routine surveillance scenario in which genetic clusters are obtained at a single or very small number of distance thresholds (e.g., thresholds for potential outbreak detection), ReporTree running times considerably decreased in all the species (also using the whole dataset) to less than 45 s with the HC algorithm and less than 1 min and 30 s with the GrapeTree algorithm (Fig. 2C), reinforcing its suitability for implementation in routine surveillance.

ReporTree represents an integrative workflow from a flexible input to a dynamic reporting of cg/wgMLST data (Fig. 1), with clustering being the central step in the analysis. As such, ReporTree offers widely used and validated algorithms (HC, goeBURST/MSTree, and MSTreeV2) for clustering purposes [4, 21]. Nevertheless, as alternative software for this step is also available, here, we compare ReporTree cgMLST clustering workflows with pHierCC [16], another commonly used method for routine surveillance and outbreak detection (implemented in Enterobase [40]). Our results (detailed in Additional file 1: Fig. S1.1 to S1.4) show that despite the conceptual differences of the tested algorithms, in general, the three clustering methods implemented in ReporTree yielded cluster number and composition highly congruent with pHierCC using the four foodborne bacteria datasets described above.

Additionally, in order to assess ReporTree performance in a different context, such as the identification of main populations (or lineages) in a dataset, we have also compared ReporTree with PopPUNK, a *k*-mer-based clustering tool that relies on machine learning to cluster genomes [41]. As recommended by the respective authors, PopPUNK was applied to assign the genomes of the *E. coli* dataset to genetic clusters using the existing *E. coli* reference database (https://www.bacpop.org/poppunk/, assessed on April 24, 2023) with the best fitting model. PopPUNK identified 366 clusters, which were found to be highly congruent with ReporTree cg/wgMLST clustering results at 642 (for MSTree and HC single-linkage) and 724 (for MSTreeV2) allele differences thresholds, with Adjusted Rand coefficients of 0.995 in both cases (Additional file 1: Fig. S2.1). These thresholds fall within one of the stability regions identified by ReporTree, reinforcing the applicability of the nAWC component of ReporTree to detect low-resolution genogroups/lineages for longitudinal surveillance or population structure evaluation.

#### Alignment-based core-SNP workflow

**Fig. 3:**
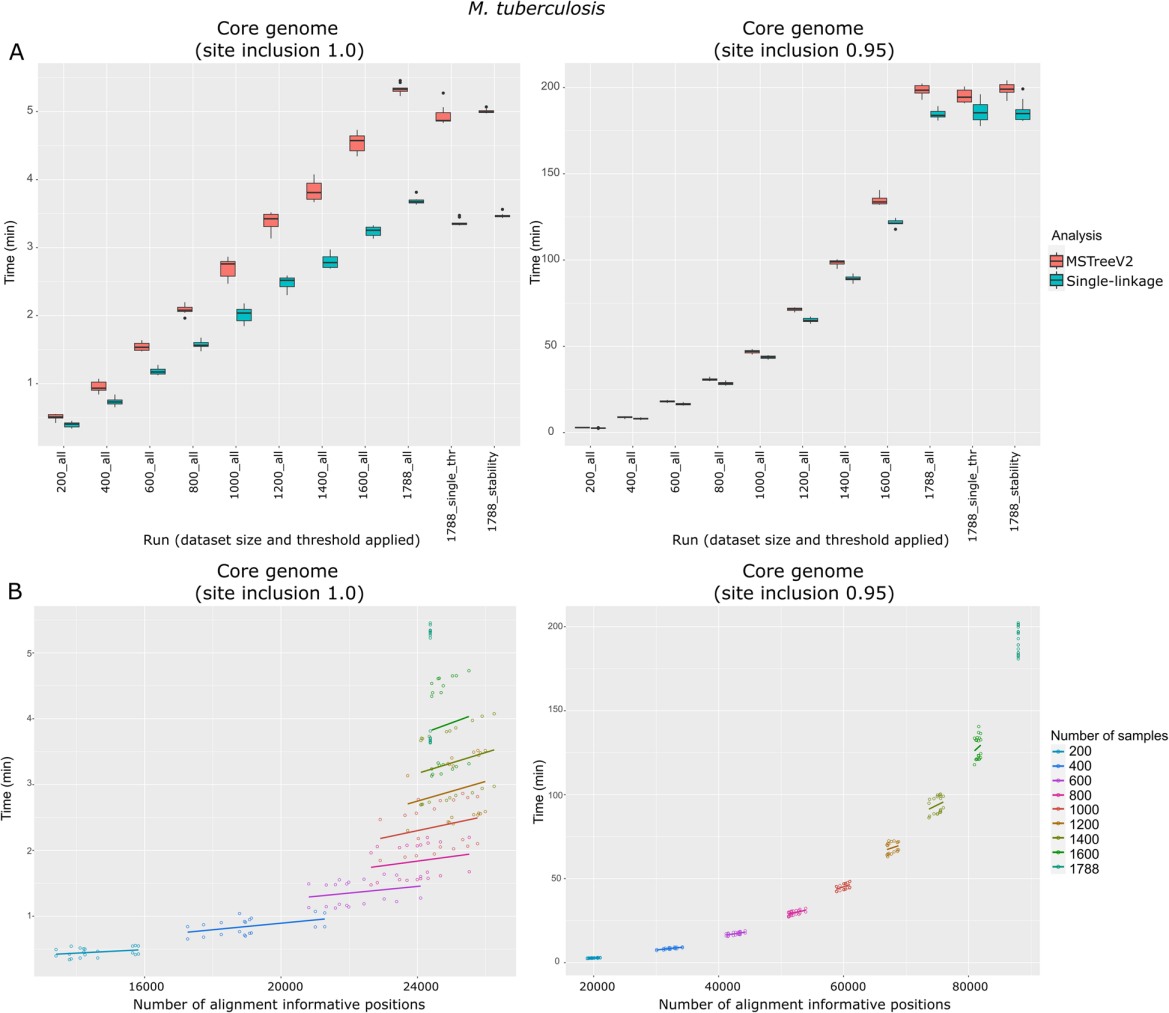
Results of ReporTree benchmarking of the alignment-based core SNP workflow using a multi-sequence alignment of 1788 *M. tuberculosis* samples and 88,562 informative nucleotide positions. **A** ReporTree running times for the 10 replicates of each sample subset with a site inclusion of 1.0 (left) and 0.95 (right), where the flag “all” indicates subsets for which ReporTree obtained clusters at all possible thresholds, the flag “single_thr” indicates subsets for which ReporTree obtained clusters at potential “transmission chain” level (12 SNP differences), and the flag “stability” indicates subsets for which ReporTree obtained clusters at all possible thresholds but only generated reports for those corresponding to stability regions. **B** ReporTree running times according to the number of variant sites obtained after alignment cleaning and that were used for clustering. Technical notes: 1. The “site-inclusion” argument defines informative nucleotide sites to be kept in the alignment based on the minimum proportion of samples per site without missing data (e.g., 1.0 reflects a “true” core alignment with all variant sites having exclusively ATCG, and 0.95 reflects a core alignment tolerating 5% of undefined nucleotides per site). 2. The *M. tuberculosis* dataset used in this benchmarking is described at [42]

ReporTree benchmarking for the alignment-based SNP workflow was performed in a laptop [Intel Core i7(R)] with 16 GB of RAM using a publicly available diverse dataset of *Mycobacterium tuberculosis* [42, 43], which is a bacterial pathogen with a large genome (approximately 4.4 Mb) for which such a workflow is starting to be routinely applied for surveillance. As input for this benchmarking, we used a filtered alignment comprising the maximum number of informative sites (a total of 88,562 nucleotide sites with at least one mutation in a given sequence) observed in the comparison of 1788 *M. tuberculosis* genomes (Fig. 3). Similar to the cg/wgMLST workflow, we generated sub-datasets of randomly selected isolates with between 200 and the maximum number of isolates in a 200-isolates step, with ten replicates per sub-dataset size, and requesting summary reports at all possible partition levels with (i) GrapeTree analysis (MSTreeV2 algorithm) and (ii) HC analysis (single-linkage algorithm). Additional runs with all isolates and requesting summary reports at potential transmission chain resolution (12 SNPs) and at stable regions were also performed. Running time was assessed using “--site-inclusion” of 1.0 (true core alignment, i.e., only ATCG) and 0.95 (core alignment tolerating 5% of undefined nucleotides per site), as a way to simulate two likely applications of ReporTree. The first one more likely suits clustering of large and diverse datasets, while the second one is more likely to be applied when fine resolution is needed (e.g., to enhance the resolution in clusters identified with less discriminatory genotyping methods).

ReporTree running time in the alignment-based core SNP analysis increases with the number of samples, linearly for “site-inclusion 1.0” and exponentially for “site-inclusion 0.95” (Fig. 3A). The considerably higher running times observed in “site-inclusion 0.95” were expected as this workflow requires a demanding cleaning step in which each variant site in the alignment has to be screened for the amount of missing data. We have also assessed how the time performance correlates with the number of informative sites used for clustering. In the “site-inclusion 1.0” workflow, for the same number of samples, having more variant sites has a minimal impact on time (Fig. 3B). On the other hand, in the “site-inclusion 0.95”, time is a function of both the number of samples and number of sites in the sense that, as expected, adding more samples introduces more variant sites to be screened for missing data. In summary, the running times were satisfactory for the main purpose of each condition, with the “site-inclusion 1.0” workflow taking less than 6 min in all tested situations and the “site-inclusion 0.95” workflow taking less than 10 min for datasets with less than 400 isolates (Fig. 3A). Of note, given the main goal of this benchmarking (considerably scale up the number of variant sites for clustering when comparing with the cg/wgMLST workflow), we used an alignment containing the maximum number of informative sites in the dataset. In a simulated scenario in which ReporTree would instead take the full genome alignment (i.e., 4.4 Mb) as input, as removing gaps and non-variable sites is less demanding than handling the “site-inclusion” argument, the time performance with the current dataset would range between 3 and 22 min for a “site-inclusion 1.0” and 12 min and 4 h and 15 min for “site-inclusion 0.95”, depending on the dataset size (200 and 1788 isolates, respectively).

The alignment-based core SNP workflow available in ReporTree is designed to facilitate the routine assessment of bacteria genetic relatedness at different levels of resolution, either as an alternative or a complement to cg/wgMLST data (e.g., to increase the resolution at outbreak level). As demonstrated in the previous benchmarkings, ReporTree relies on widely used and validated methods that ensure input flexibility, clustering reliability, and turn-around times compatible with routine purposes. Still, core SNP-based analyses are also often applied for the identification of main bacterial populations (or lineages) towards the reconstruction of pathogen evolutionary history or species-level population structure. As such, we sought to assess how ReporTree-derived clustering compares with the lineages/populations obtained through a traditional typing method and also through a Bayesian analysis of population structure (BAPS). To this end, we not only took advantage of the lineage information of the *M. tuberculosis* dataset (inferred using Tb-profiler v4.4.1. [44]) but also ran FastBAPS [45] on its multi-sequence alignment using default parameters (details in Additional file 1). Similar to the cg/wgMLST benchmarking (previous subsection), clusters were identified at all possible thresholds with ReporTree using MSTreeV2 method. We found that the levels with highest congruence either with *M. tuberculosis* main lineages or with FastBAPS fall within ReporTree-determined stability regions for this dataset (details in Additional file 1), again demonstrating the alternative utility of ReporTree to get insight on bacterial population structure.

### Reproducing a large-scale study on genetic clustering and linkage to antibiotic resistance data in *Neisseria gonorrhoeae*

Our team has recently performed an extensive genomics analysis of the bacterial pathogen *Neisseria gonorrhoeae* [46]. In this study, 3791 *N. gonorrhoeae* genomes from isolates collected across Europe were analyzed with a cgMLST approach. Genetic clusters were determined with the goeBURST algorithm implemented in PHYLOViZ [3, 12, 18, 47] for all possible allelic distance thresholds (partitions). Cluster concordance between subsequent distance thresholds was assessed with the nAWC in order to determine regions of cluster stability [14, 22, 25, 26] that were used for nomenclature purposes and identification of genogroups. The association between metadata and genetic clusters was then performed by time-consuming table handling with a spreadsheet program. This corresponded to a non-automated workflow and, in the particular case of the cluster congruence analysis and the integration of genetic and clinically or epidemiologically relevant data, it represented a highly demanding process difficult to be applied in real-time pathogen surveillance. As such, to validate ReporTree and demonstrate how it can enhance bacterial pathogens’ surveillance and research, we used the same dataset as in the previous study [46] and attempted to reproduce the main study outputs with this tool. As shown in ReporTree’s Wiki [48], using the allele matrix with 822 loci [49] and the associated metadata (available in Supplementary material 1 of Pinto et al. [46]) as input, ReporTree automatically identified the genetic clusters at all possible partition thresholds of the generated MST, identified the same regions of cluster stability, and replicated the hierarchical nomenclature applied by Pinto et al. [46]. Moreover, it provided an updated metadata table with clustering information at the first partition of each stability region and of the derived nomenclature code, which could be used as input for visualization in GrapeTree [4]. Furthermore, summary reports with statistics/trends associated with each genetic cluster of low and high levels of stability (i.e., 40 allele differences at the lower level and 79 allele differences at the higher level, similarly to what was found by Pinto et al.) were reported. Of note, the high level of stability identified by ReporTree matches the lineages identified by PopPUNK with the same dataset (details in Additional file 1: Fig. S2.2), supporting that this resolution level reflects *N. gonorrhoeae* population structure. Finally, ReporTree was able to associate and report the distribution of genetic determinants of antimicrobial resistance in *N. gonorrhoeae* for the different genetic clusters. Importantly, this example allowed a clear validation of the tool by rigorously reproducing the data presented, for example, in Figure 1a, 1b and 3 and in Tables 1 and 2 of the previous publication [46]. All these outputs (and additional ones) are available for consultation at ReporTree github repository [28]. Noteworthy, this proof of concept was made with a single ReporTree command line that ran for approximately 2 min and 2 s in a laptop [Intel Core i5(R)] with 16 GB of RAM.

### ReporTree and its application to genomics-informed routine surveillance (e.g., SARS-CoV-2) and outbreak detection (e.g., *Listeria monocytogenes*)

ReporTree’s versatility and broad functionalities make it suitable for different applications and integration in different research or surveillance contexts (from the usage of specific functionalities to the whole pipeline implementation). Here, we provide two examples of the integration of this tool, at different scales, in established workflows of genomic surveillance.

Genomics-informed surveillance of SARS-CoV-2 has had an important role in worldwide public health and political decision-making in the last 2 years. In Portugal, weekly reports of nationwide sequencing surveys are provided to public health authorities and the general public describing important indicators and trends of the evolution and geotemporal spread of the virus [50]. Therefore, after ReporTree validation, we implemented this tool in the routine genomics surveillance of SARS-CoV-2 in the country with the objective of speeding up the association between genomic and epidemiological data and the generation of the surveillance-oriented reports. For instance, besides its comprehensive usage for calculating the relative frequency of variants of concern (VOCs) at regional and national levels, ReporTree is often applied to identify clusters of high-closely related viruses (e.g., using TreeCluster [8] max-clade or avg-clade models at high resolution levels) that may represent local transmission networks or even super-spreading events. Examples of ReporTree application in the context of SARS-CoV-2 genomic epidemiology are provided at ReporTree’s Wiki [48]. To further demonstrate its utility and performance for this purpose, we ran ReporTree over the public Taxonium (https://cov2tree.org/) [7] tree with more than 6 million SARS-CoV-2 sequences (details in Additional file 1). ReporTree is able to cut this massive tree, identifying all clusters of close-related sequences (e.g., avg-clade at 2 SNP differences), and extract valuable information (timespan, country dispersion, etc.) for the clusters identified in a given lineage, country, or period of time.

Regarding the full implementation, one of the most direct and intuitive applications is the analysis of cg/wgMLST data for outbreak investigation, namely for foodborne bacterial pathogens (as shown in the Benchmarking section), as this subtyping method delivers sufficiently high-resolution and epidemiological concordance [51]. In ReporTree’s Wiki [48], it is provided a simple simulated example in which, with a single command line, ReporTree builds an MST from cgMLST data and automatically extracts and reports genetic clusters of *L. monocytogenes* at high-resolution levels commonly used for outbreak detection (≤ 4 and ≤ 7 allelic differences, [39]), keeping the cluster nomenclature of the previous run, as routinely performed in Portugal. These two examples show that ReporTree is a useful asset to rapidly generate summary reports with key data (pathogen genetic clusters) and statistics/trends for routine surveillance and outbreak investigation.

## Conclusions

ReporTree represents an automated and flexible pipeline that can be used for a wide variety of species and that facilitates the detection of genetic clusters and their linkage to epidemiological data, in a concept aligned with “One Health” perspectives. Here, we presented the proof of concept of this tool, showing its ability to quickly report a comprehensive WGS-based genogroup assignment for *N. gonorrhoeae*, based on the identification of the discriminatory genetic thresholds reflecting cluster stability, and the rapid correlation of these genogroups (representing main circulating lineages) with any data of interest, such as antimicrobial resistance data. Furthermore, we have shown how its flexibility contributed to speed up SARS-CoV-2 and *L. monocytogenes* genomics-informed surveillance in Portugal, facilitating and accelerating the production of surveillance-oriented reports. ReporTree benchmarking ultimately demonstrated that this tool can be smoothly implemented in routine surveillance bioinformatics workflows, with negligible computational and time costs. Although ReporTree is currently available as a command line tool, this resource can easily be integrated in start-to-end platforms for genomics/epidemiological analysis (for instance, it will be soon integrated in the COHESIVE Information System [52] and INSaFLU platform [53]), thus contributing to a sustainable and efficient public health genomics-informed pathogen surveillance.

## Availability and requirements

Project name: ReporTree.

Project home page: https://github.com/insapathogenomics/ReporTree

Project Wiki: https://github.com/insapathogenomics/ReporTree/wiki

Record of the home page and Wiki at the time of publication: https://doi.org/10.5281/zenodo.7772640

Operating system(s): Unix.

Programming language: Python 3.8

Other requirements: Biopython 1.77, Pandas 1.1.3, Ete3, TreeCluster 1.0.3, GrapeTree 2.1, cgmlst-dists, and vcf2mst.

License: GPL 3.0

Any restrictions to use by non-academics: none.

## Supplementary Information

**Additional file 1.** Comparison of ReporTree results with other clustering methods and tools.

## Data Availability

The datasets generated and/or analyzed during the current study are available in the Zenodo repository (*L. monocytogenes*: https://zenodo.org/record/7116879 [31]; *S. enterica*: https://zenodo.org/record/7119736 [32]; *E. coli*: https://zenodo.org/record/7120058 [33]; *C. jejuni*: https://doi.org/10.5281/zenodo.7120167 [34]; *N. gonorrhoeae*: https://zenodo.org/record/3946223 [49]; *M. tuberculosis*: https://zenodo.org/record/7772652 [43]) or in ReporTree github repository at https://github.com/insapathogenomics/ReporTree/ [28]. In order to ensure the long-term availability of all materials used in this manuscript, the github repository and the Wiki material at the time of publication are also available at Zenodo (https://doi.org/10.5281/zenodo.7772640) [29].

## Abbreviations

cgMLST: Core-genome multilocus sequence type
HC: Hierarchical clustering
MST: Minimum spanning tree
nAWC: Neighborhood Adjusted Wallace coefficient
SNP: Single-nucleotide polymorphism
wgMLST: Whole-genome multilocus sequence type
WGS: Whole-genome sequencing

## References

[1] Jolley KA, Maiden MCJ (2014). Using multilocus sequence typing to study bacterial variation: prospects in the genomic era. Future Microbiol.

[2] Wohl S, Schaffner SF, Sabeti PC (2016). Genomic analysis of viral outbreaks. Annu Rev Virol.

[3] Ribeiro-Gonçalves B, Francisco AP, Vaz C, Ramirez M, Carriço JA (2016). PHYLOViZ Online: web-based tool for visualization, phylogenetic inference, analysis and sharing of minimum spanning trees. Nucleic Acids Res.

[4] Zhou Z, Alikhan N-F, Sergeant MJ, Luhmann N, Vaz C, Francisco AP (2018). GrapeTree: visualization of core genomic relationships among 100,000 bacterial pathogens. Genome Res.

[5] Hadfield J, Megill C, Bell SM, Huddleston J, Potter B, Callender C (2018). Nextstrain: real-time tracking of pathogen evolution. Bioinformatics.

[6] Argimón S, Abudahab K, Goater RJE, Fedosejev A, Bhai J, Glasner C (2016). Microreact: visualizing and sharing data for genomic epidemiology and phylogeography. Microb Genom.

[7] Sanderson T (2022). Taxonium, a web-based tool for exploring large phylogenetic trees. eLife.

[8] Balaban M, Moshiri N, Mai U, Jia X, Mirarab S (2019). TreeCluster: clustering biological sequences using phylogenetic trees. PLoS one.

[9] Dallman T, Ashton P, Schafer U, Jironkin A, Painset A, Shaaban S (2018). SnapperDB: a database solution for routine sequencing analysis of bacterial isolates. Bioinformatics.

[10] Deneke C, Uelze L, Brendebach H, Tausch SH, Malorny B (2021). Decentralized investigation of bacterial outbreaks based on hashed cgMLST. Front Microbiol.

[11] Ragonnet-Cronin M, Hodcroft E, Hué S, Fearnhill E, Delpech V, Brown AJL (2013). Automated analysis of phylogenetic clusters. BMC Bioinformatics.

[12] Francisco AP, Vaz C, Monteiro PT, Melo-Cristino J, Ramirez M, Carriço JA (2012). PHYLOViZ: phylogenetic inference and data visualization for sequence based typing methods. BMC Bioinformatics.

[13] Lees JA, Harris SR, Tonkin-Hill G, Gladstone RA, Lo SW, Weiser JN (2019). Fast and flexible bacterial genomic epidemiology with PopPUNK. Genome Res.

[14] Llarena A-K, Ribeiro-Gonçalves BF, Nuno Silva D, Halkilahti J, Machado MP, Da Silva MS (2018). INNUENDO: a cross-sectoral platform for the integration of genomics in the surveillance of food-borne pathogens. EFSA Support Publ.

[15] Rambaut A, Holmes EC, O’Toole Á, Hill V, McCrone JT, Ruis C (2020). A dynamic nomenclature proposal for SARS-CoV-2 lineages to assist genomic epidemiology. Nat Microbiol.

[16] Zhou Z, Charlesworth J, Achtman M (2021). HierCC: a multi-level clustering scheme for population assignments based on core genome MLST. Bioinformatics.

[17] Di Pasquale A, Radomski N, Mangone I, Calistri P, Lorusso A, Cammà C (2021). SARS-CoV-2 surveillance in Italy through phylogenomic inferences based on Hamming distances derived from pan-SNPs, -MNPs and -InDels. BMC Genomics.

[18] Francisco AP, Bugalho M, Ramirez M, Carriço JA (2009). Global optimal eBURST analysis of multilocus typing data using a graphic matroid approach. BMC Bioinformatics.

[19] GrapeTree (github repository with the modified version). https://github.com/insapathogenomics/GrapeTree (open page continuously updated).

[20] Seemann T. cgmlst-dists. github. https://github.com/tseemann/cgmlst-dists Accessed on 28 Sept 2022.

[21] Virtanen P, Gommers R, Oliphant TE, Haberland M, Reddy T, Cournapeau D (2020). SciPy 1.0: fundamental algorithms for scientific computing in Python. Nat Methods.

[22] Carriço JA, Silva-Costa C, Melo-Cristino J, Pinto FR, de Lencastre H, Almeida JS (2006). Illustration of a common framework for relating multiple typing methods by application to macrolide-resistant Streptococcus pyogenes. J Clin Microbiol.

[23] Comparing partitions. http://www.comparingpartitions.info/ Accessed on 28 Sept 2022.

[24] Comparing partitions (repository with the new version). Github. https://github.com/insapathogenomics/ComparingPartitions Accessed on 28 Sept 2022.

[25] Severiano A, Pinto FR, Ramirez M, Carriço JA (2011). Adjusted Wallace coefficient as a measure of congruence between typing methods. J Clin Microbiol.

[26] Barker DOR, Carriço JA, Kruczkiewicz P, Palma F, Rossi M, Taboada EN. Rapid identification of stable clusters in bacterial populations using the adjusted Wallace coefficient bioRxiv. 2018. Available from: http://biorxiv.org/lookup/doi/10.1101/299347.

[27] Di Pasquale A, Radomski N, Maassen K, Cammà C. One Health structure In Europe for omics-based surveillance. Available from: https://github.com/genpat-it/grapetree-gis.

[28] ReporTree github. https://github.com/insapathogenomics/ReporTree (open page continuously updated).

[29] Mixão V, Pinto M, Sobral D, Di Pasquale A, Gomes JP, Borges V. ReporTree: a surveillance-oriented tool to strengthen the linkage between pathogen genetic clusters and epidemiological data. 2023. Zenodo. 10.5281/zenodo.7772640. 10.1186/s13073-023-01196-1 PMC10273728 37322495

[30] ReporTree (docker). https://hub.docker.com/r/insapathogenomics/reportree (open page continuously updated).

[31] Mixão V, Brendebach H, Pinto M, Sobral D, Gomes JP, Deneke C, et al. Genome assemblies and respective cgMLST profiles of a diverse dataset comprising 1,874 *Listeria monocytogenes* isolates. Zenodo. 2022. https://zenodo.org/record/7116879.

[32] Mixão V, Brendebach H, Pinto M, Sobral D, Gomes JP, Deneke C, et al. Genome assemblies and respective wg/cgMLST profiles of a diverse dataset comprising 1,434 *Salmonella enterica* isolates. Zenodo. 2022. https://zenodo.org/record/7119736.

[33] Mixão V, Brendebach H, Pinto M, Sobral D, Gomes JP, Deneke C, et al. Genome assemblies and respective wg/cgMLST profiles of a diverse dataset comprising 1,999 *Escherichia coli* isolates. Zenodo. 2022. https://zenodo.org/record/7120058.

[34] Mixão V, Brendebach H, Pinto M, Sobral D, Gomes JP, Deneke C, et al. Genome assemblies and respective wg/cgMLST profiles of a diverse dataset comprising 3,076 *Campylobacter jejuni* isolates. Zenodo. 2022. https://zenodo.org/record/7120167.

[35] Deneke C, Brendebach H, Uelze L, Borowiak M, Malorny B, Tausch SH (2021). Species-specific quality control, assembly and contamination detection in microbial isolate sequences with AQUAMIS. Genes.

[36] Silva M, Machado MP, Silva DN, Rossi M, Moran-Gilad J, Santos S (2018). chewBBACA: a complete suite for gene-by-gene schema creation and strain identification. Microb Genom.

[37] Moura A, Criscuolo A, Pouseele H, Maury MM, Leclercq A, Tarr C (2016). Whole genome-based population biology and epidemiological surveillance of *Listeria monocytogenes*. Nat Microbiol.

[38] Mamede R, Vila-Cerqueira P, Silva M, Carriço JA, Ramirez M (2021). Chewie Nomenclature Server (chewie-NS): a deployable nomenclature server for easy sharing of core and whole genome MLST schemas. Nucleic Acids Res.

[39] Van Walle I, Björkman JT, Cormican M, Dallman T, Mossong J, Moura A (2018). Retrospective validation of whole genome sequencing-enhanced surveillance of listeriosis in Europe, 2010 to 2015. Euro Surveill.

[40] Zhou Z, Alikhan N-F, Mohamed K, Fan Y, Achtman M, Agama Study Group (2020). The EnteroBase user’s guide, with case studies on Salmonella transmissions, Yersinia pestis phylogeny, and Escherichia core genomic diversity. Genome Res.

[41] Lees JA, Harris SR, Tonkin-Hill G, Gladstone RA, Lo SW, Weiser JN (2019). Fast and flexible bacterial genomic epidemiology with PopPUNK. Genome Res.

[42] Walker TM, Kohl T, Omar SV, Hedge J, Elias CDO, Bradley P (2015). Whole-genome sequencing for prediction of *Mycobacterium tuberculosis* drug susceptibility and resistance: a retrospective study. Lancet Infect Dis.

[43] Mixão V, Pinto M, Sobral D, Di Pasquale A, Gomes JP, Borges V. Multiple sequence alignment of a diverse dataset with 1788 *Mycobacterium tuberculosis* isolates. Zenodo. 2023. https://zenodo.org/record/7772652.

[44] Phelan JE, O'Sullivan DM, Machado D, Ramos J, Oppong YEA, Campino S (2019). Integrating informatics tools and portable sequencing technology for rapid detection of resistance to anti-tuberculous drugs. Genome Med.

[45] Tonkin-Hill G, Lees JA, Bentley SD, Frost SDW, Corander J (2019). Fast hierarchical Bayesian analysis of population structure. Nucleic Acids Res.

[46] Pinto M, Borges V, Isidro J, Rodrigues JC, Vieira L, Borrego MJ (2021). *Neisseria gonorrhoeae* clustering to reveal major European whole-genome-sequencing-based genogroups in association with antimicrobial resistance. Microb Genom.

[47] Nascimento M, Sousa A, Ramirez M, Francisco AP, Carriço JA, Vaz C (2017). PHYLOViZ 2.0: providing scalable data integration and visualization for multiple phylogenetic inference methods. Bioinformatics..

[48] ReporTree Wiki. https://github.com/insapathogenomics/ReporTree/wiki (open page continuously updated).

[49] Pinto M, Borges V, Isidro J, Rodrigues JC, Vieira L, Borrego MJ, et al. *Neisseria gonorrhoeae* clustering to reveal major European WGS-based genogroups in association with antimicrobial resistance (cgMLST and MScgMLST schemas, allelic profile matrices and GrapeTree input file). Zenodo. 2020. https://zenodo.org/record/3946223.

[50] Diversidade genética do novo coronavírus SARS-CoV-2 (COVID-19) em Portugal. Available from: https://insaflu.insa.pt/covid19/ Accessed on 12 May 2023.

[51] Nadon C, Van Walle I, Gerner-Smidt P, Campos J, Chinen I, Concepcion-Acevedo J (2017). PulseNet International: vision for the implementation of whole genome sequencing (WGS) for global food-borne disease surveillance. Euro Surveill.

[52] Sciensano. D3.12- abstract book for 2nd annual scientific meeting (ASM). Zenodo; 2021. https://zenodo.org/record/4897305.

[53] Borges V, Pinheiro M, Pechirra P, Guiomar R, Gomes JP (2018). INSaFLU: an automated open web-based bioinformatics suite “from-reads” for influenza whole-genome-sequencing-based surveillance. Genome Med.

