# Supplementary material for: ReporTree: a surveillance-oriented tool to strengthen the linkage between pathogen genetic clusters and epidemiological data
Source: Genome Med. 2023 Jun 15;15:43. doi: 10.1186/s13073-023-01196-1 (PMC10273728; doi:10.1186/s13073-023-01196-1)
Supplement: Supplementary file 1 — Additional file 1. Comparison of ReporTree results with other clustering methods and tools. [file 13073_2023_1196_MOESM1_ESM.docx]

**Additional file 1. Comparison of ReporTree results with other clustering methods and tools.**

This file includes the clustering results obtained by ReporTree when compared to other tools. It is divided into different exercises that cover different goals, pathogens and input types.

**Exercise 1**

Goal: Assess how cg/wgMLST ReporTree-derived clustering compares with pHierCC at all possible levels of resolution for surveillance purposes

Datasets: *Listeria monocytogenes* (1,874 isolates: <https://zenodo.org/record/7116879>); *Salmonella enterica* (1,434 isolates: <https://zenodo.org/record/7119736>); *Escherichia coli* (1,999 isolates: <https://zenodo.org/record/7120058>); *Campylobacter jejuni* (3,076 isolates: <https://zenodo.org/record/7120058>)

Input: cgMLST allele matrix (filtered out samples with <95% loci called)

Software settings:

*pHierCC (*[*https://github.com/zheminzhou/pHierCC*](https://github.com/zheminzhou/pHierCC)*)*

version 1.24 (default settings)

*ReporTree (*[*https://github.com/insapathogenomics/ReporTree*](https://github.com/insapathogenomics/ReporTree)*)*

version 2.0.0 (default settings) using the following clustering methods:

i) hierarchical clustering (single-linkage)

ii) MSTree (goeBURST)

iii) MSTreeV2 (GrapeTree)

Technical notes:

- All runs were performed in a laptop [Intel Core i7(R)] with 16 GB of RAM using a single thread.
- The running times only cover the ReporTree step under comparison in this exercise, i.e. the clustering.
- Adjusted Rand was performed using comparing_partitions_v2.py (default settings): <https://github.com/insapathogenomics/ComparingPartitions>
- Adjusted Rand coefficient indicates the concordance of two typing methods, or, in this case, of two partitions of different methods (it varies between 0 and 1, 1 representing full concordance)

Results:


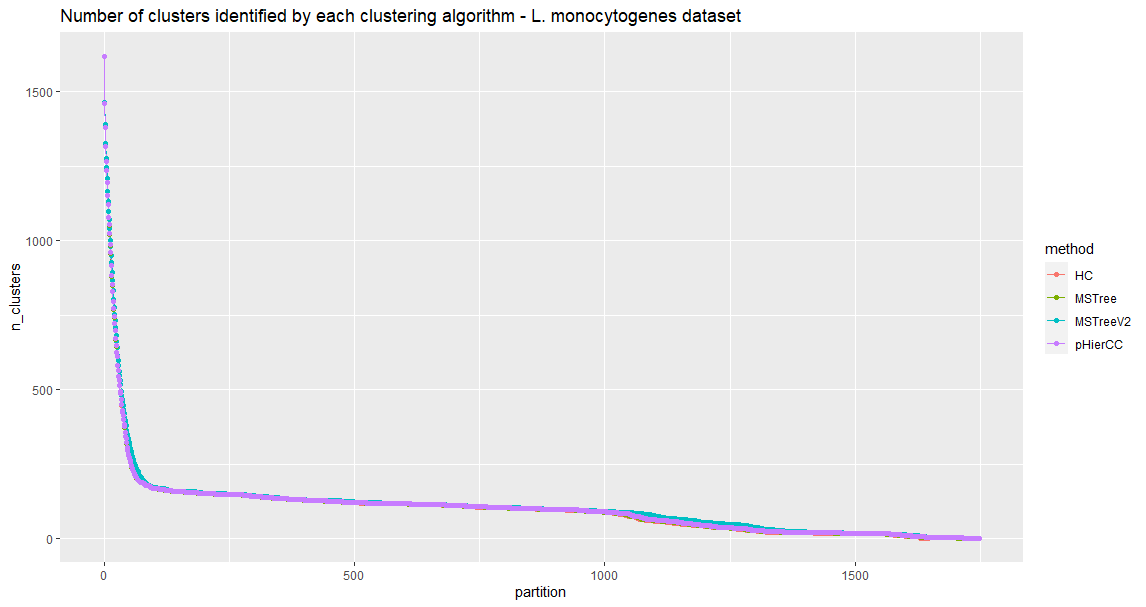


**Fig. S1.1.** cgMLST ReporTree clustering versus pHierCC, using the *L. monocytogenes* dataset. Comparison of the clustering results obtained with the HC, MSTree and MSTreeV2 method implemented in ReporTree with pHierCC, with indication of the number of clusters obtained by the different methods at each possible distance threshold.

**Table S1.1:** Comparison of ReporTree clustering methods and pHierCC for the *L. monocytogenes* dataset (cgMLST schema with 1748 loci), assessed by the number of clusters and the Adjusted Rand coefficient (presented between parentheses) obtained at incremental allele distance (AD) thresholds.

|  | ***Number of clusters***  ***(Adjusted Rand coefficient versus pHierCC)*** | | | |
| --- | --- | --- | --- | --- |
| **Threshold**  **[% schema (AD)]** | **pHierCC** | **ReporTree (HC)** | **ReporTree**  **(MSTree)** | **ReporTree**  **(MSTreeV2)** |
| 0.4 % (7 ADs)* | 1053 (1.0) | 1050 (0.99) | 1050 (0.99) | 1072 (0.93) |
| 1 % (17 ADs) | 829 (1.0) | 828 (0.99) | 828 (0.99) | 865 (0.88) |
| 5 % (87 ADs) | 179 (1.0) | 179 (1.0) | 179 (1.0) | 182 (0.99) |
| 10 % (145 ADs) | 158 (1.0) | 158 (1.0) | 158 (1.0) | 158 (1.0) |
| 50 % (874 ADs) | 99 (1.0) | 99 (1.0) | 99 (1.0) | 100 (0.99) |

** potential “outbreak” level as applied in the benchmarking section of the manuscript (Figure 2)*

**
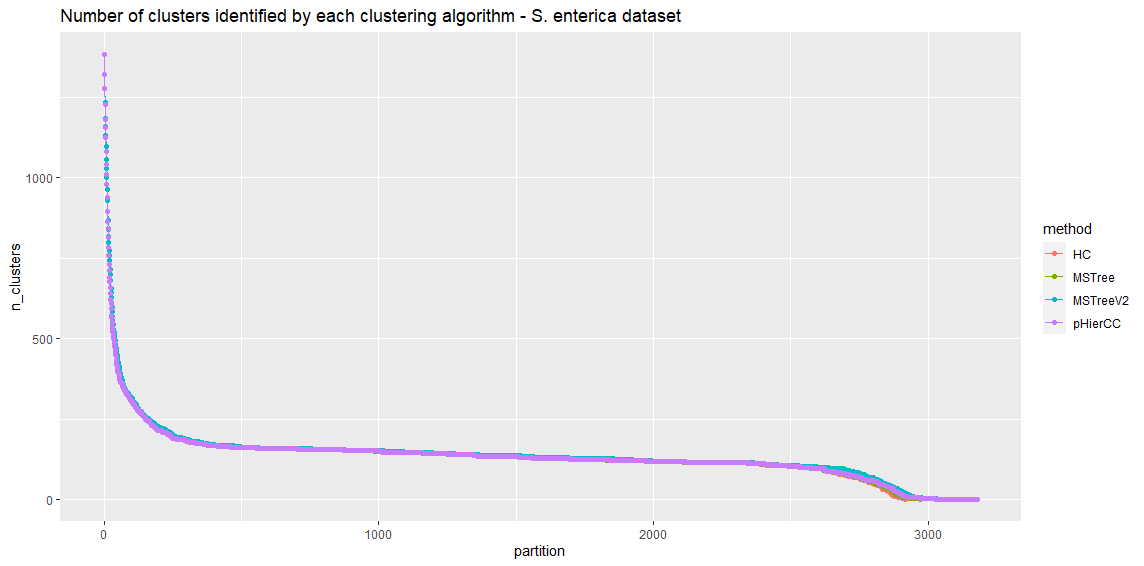
**

**Fig. S1.2.** cgMLST ReporTree clustering versus pHierCC, using the *S. enterica* dataset. Comparison of the clustering results obtained with the HC, MSTree and MSTreeV2 method implemented in ReporTree with pHierCC, with indication of the number of clusters obtained by the different methods at each possible distance threshold.

**Table S1.2:** Comparison of ReporTree clustering methods and pHierCC for the *S. enterica* dataset (cgMLST schema with 3179 loci), assessed by the number of clusters and the Adjusted Rand coefficient (presented between parentheses) obtained at incremental allele distance (AD) thresholds.

|  | ***Number of clusters***  ***(Adjusted Rand coefficient versus pHierCC)*** | | | |
| --- | --- | --- | --- | --- |
| **Threshold**  **[% schema (AD)]** | **pHierCC** | **ReporTree (HC)** | **ReporTree**  **(MSTree)** | **ReporTree**  **(MSTreeV2)** |
| 0.43 % (14 ADs)* | 842 (1.0) | 842 (1.0) | 842 (1.0) | 866 (0.73) |
| 1 % (32 ADs) | 524 (1.0) | 522 (0.99) | 522 (0.99) | 556 (0.92) |
| 5 % (159 ADs) | 246 (1.0) | 246 (1.0) | 246 (1.0) | 251 (0.99) |
| 10 % (318 ADs) | 176 (1.0) | 176 (1.0) | 176 (1.0) | 181 (0.75) |
| 50 % (1590 ADs) | 129 (1.0) | 129 (1.0) | 129 (1.0) | 132 (0.99) |

** potential “outbreak” level as applied in the benchmarking section of the manuscript (Figure 2)*


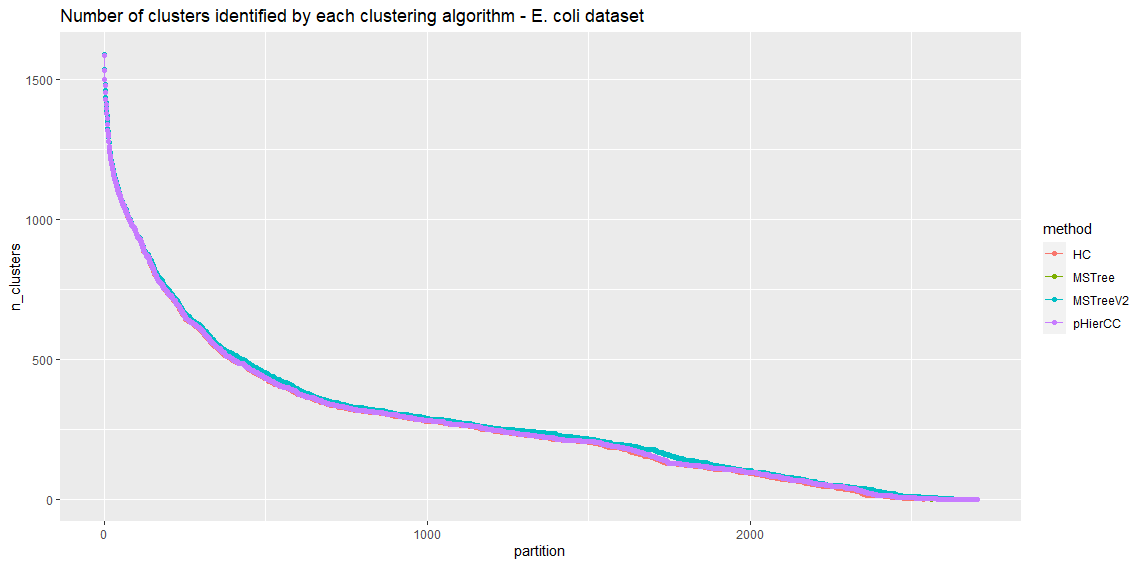


**Fig. S1.3.** cgMLST ReporTree clustering versus pHierCC, using the *E. coli* dataset. Comparison of the clustering results obtained with the HC, MSTree and MSTreeV2 method implemented in ReporTree with pHierCC, with indication of the number of clusters obtained by the different methods at each possible distance threshold.

**Table S1.3:** Comparison of ReporTree clustering methods and pHierCC for the *E. coli* dataset (cgMLST schema with 2704 loci), assessed by the number of clusters and the Adjusted Rand coefficient (presented between parentheses) obtained at incremental allele distance (AD) thresholds.

|  | ***Number of clusters***  ***(Adjusted Rand coefficient versus pHierCC)*** | | | |
| --- | --- | --- | --- | --- |
| **Threshold**  **[% schema (AD)]** | **pHierCC** | **ReporTree (HC)** | **ReporTree**  **(MSTree)** | **ReporTree**  **(MSTreeV2)** |
| 0.34 % (9 ADs)* | 1362 (1.0) | 1362 (1.0) | 1362 (1.0) | 1370 (0.97) |
| 1 % (27 ADs) | 1178 (1.0) | 1178 (1.0) | 1178 (1.0) | 1185 (0.98) |
| 5 % (135 ADs) | 867 (1.0) | 867 (1.0) | 867 (1.0) | 875 (0.96) |
| 10 % (270 ADs) | 633 (1.0) | 633 (1.0) | 633 (1.0) | 643 (0.99) |
| 50 % (1352 ADs) | 225 (1.0) | 224 (1.0) | 223 (1.0) | 239 (0.98) |

** potential “outbreak” level as applied in the benchmarking section of the manuscript (Figure 2)*


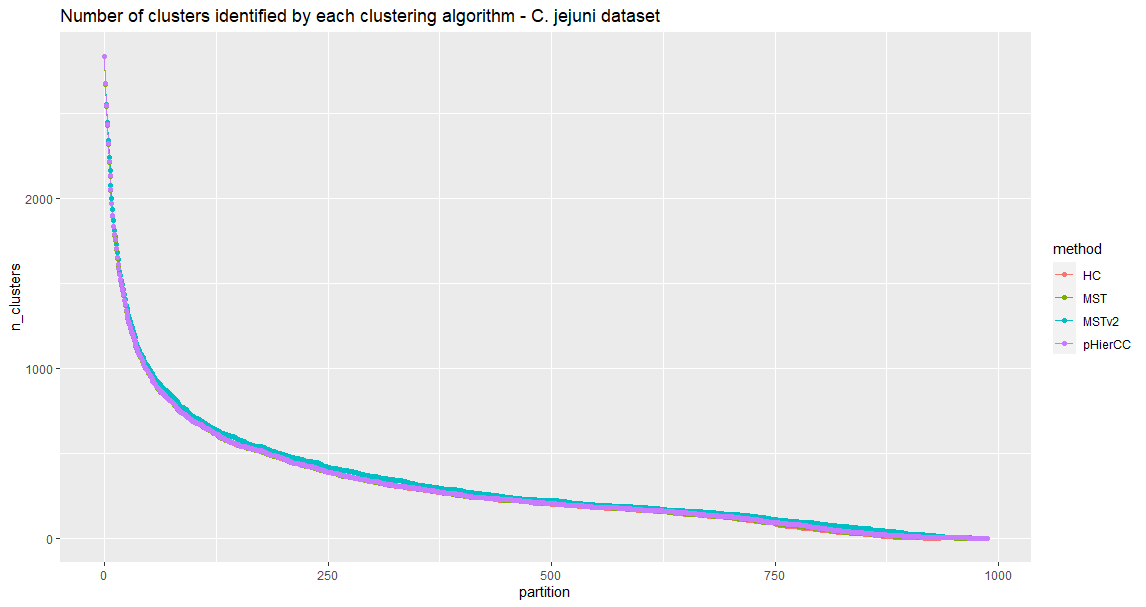


**Fig. S1.4.** cgMLST ReporTree clustering versus pHierCC, using the *C. jejuni* dataset. Comparison of the clustering results obtained with the HC, MSTree and MSTreeV2 method implemented in ReporTree with pHierCC, with indication of the number of clusters obtained by the different methods at each possible distance threshold.

**Table S1.4:** Comparison of ReporTree clustering methods and pHierCC for the *C. jejuni* dataset (cgMLST schema with 987 loci), assessed by the number of clusters and the Adjusted Rand coefficient (presented between parentheses) obtained at incremental allele distance (AD) thresholds.

|  | ***Number of clusters***  ***(Adjusted Rand coefficient versus pHierCC)*** | | | |
| --- | --- | --- | --- | --- |
| **Threshold**  **[% schema (AD)]** | **pHierCC** | **ReporTree (HC)** | **ReporTree**  **(MSTree)** | **ReporTree**  **(MSTreeV2)** |
| 0.59 % (6 ADs)* | 2137 (1.0) | 2134 (0.99) | 2134 (0.99) | 2165 (0.91) |
| 1 % (10 ADs) | 1840 (1.0) | 1836 (0.99) | 1836 (0.99) | 1873 (0.92) |
| 5 % (49 ADs) | 977 (1.0) | 972 (0.99) | 973 (0.99) | 1008 (0.87) |
| 10 % (99 ADs) | 689 (1.0) | 587 (0.99) | 687 (0.99) | 716 (0.96) |
| 50 % (494 ADs) | 209 (1.0) | 206 (0.97) | 208 (0.99) | 225 (0.73) |

** potential “outbreak” level as applied in the benchmarking section of the manuscript (Figure 2)*

Interpretation:

These results show that the three methods implemented in ReporTree, namely, HC, MSTree and MSTreeV2, all of them broadly used in the genomics surveillance field, provide congruent results with pHierCC (another common clustering solution, implemented in Enterobase). This was observed across the four different datasets of foodborne bacterial pathogens, with HC and MSTree being consistently more concordant with pHierCC than MSTreeV2, which most likely reflects the conceptual differences between the algorithms. This exercise demonstrates that ReporTree provides a set of clustering solutions suitable for application in multiple pathogens and scenarios, from outbreak-investigation to longitudinal genomics surveillance.

**Table S1.5:** Running times of each of the clustering methods used in Exercise 1 in seconds.

|  | ***L. monocytogenes***  **(s)** | ***S. enterica***  ***(s)*** | ***E. coli***  ***(s)*** | ***C. jejuni***  ***(s)*** |
| --- | --- | --- | --- | --- |
| **ReporTree (MSTreeV2)** | 65 | 79 | 113 | 105 |
| **ReporTree (MSTree)** | 59 | 73 | 92 | 94 |
| **ReporTree**  **(HC)** | 33 | 46 | 71 | 47 |
| **pHierCC** | 12 | 12 | 19 | 18 |

**Exercise 2**

Goal: Assess how cgMLST ReporTree-derived clustering compares with the lineages/populations obtained by the *K*-mer-based approach of PopPUNK

Datasets: *E. coli*: <https://zenodo.org/record/7120058> and *N. gonorrhoeae*: <https://zenodo.org/record/3946223> (only the cgMLST datasets used in this publication for which a PopPUNK database was available and functional were used for the exercise. Note: An issue in the PopPUNK database for *L. monocytogenes* hampered its usage at the time of publication)

Input: Assemblies (PopPUNK) and cgMLST allele matrix (ReporTree)

Software settings:

*PopPUNK (*[*https://www.poppunk.net/*](https://www.poppunk.net/)*)*

version 2.6.0 (default settings)

Databases:

- *E. coli* v2 (reference database)
- *N. gonorrhoeae* v1 (reference database)

*ReporTree (*[*https://github.com/insapathogenomics/ReporTree*](https://github.com/insapathogenomics/ReporTree)*)*

version 2.0.0 (default settings with the exception of “--partitions2report stability_regions”) using the following clustering methods:

i) hierarchical clustering (single-linkage)

ii) MSTree (goeBURST)

iii) MSTreeV2 (GrapeTree)

Technical notes:

- All runs were performed in a laptop [Intel Core i7(R)] with 16 GB of RAM using a single thread.
- The running times only cover the ReporTree step under comparison in this exercise, i.e. the clustering
- As we used PopPUNK available databases, clustering with PopPUNK was performed with a single command line using the poppunk_assign option (default settings).
- As both tools have different starting points, comparison of their running times should be made with caution.
- Adjusted Rand calculation was performed using comparing_partitions_v2.py (default settings): <https://github.com/insapathogenomics/ComparingPartitions>
- Adjusted Rand coefficient indicates the concordance of two typing methods (it varies between 0 and 1, 1 representing full concordance)

Results:


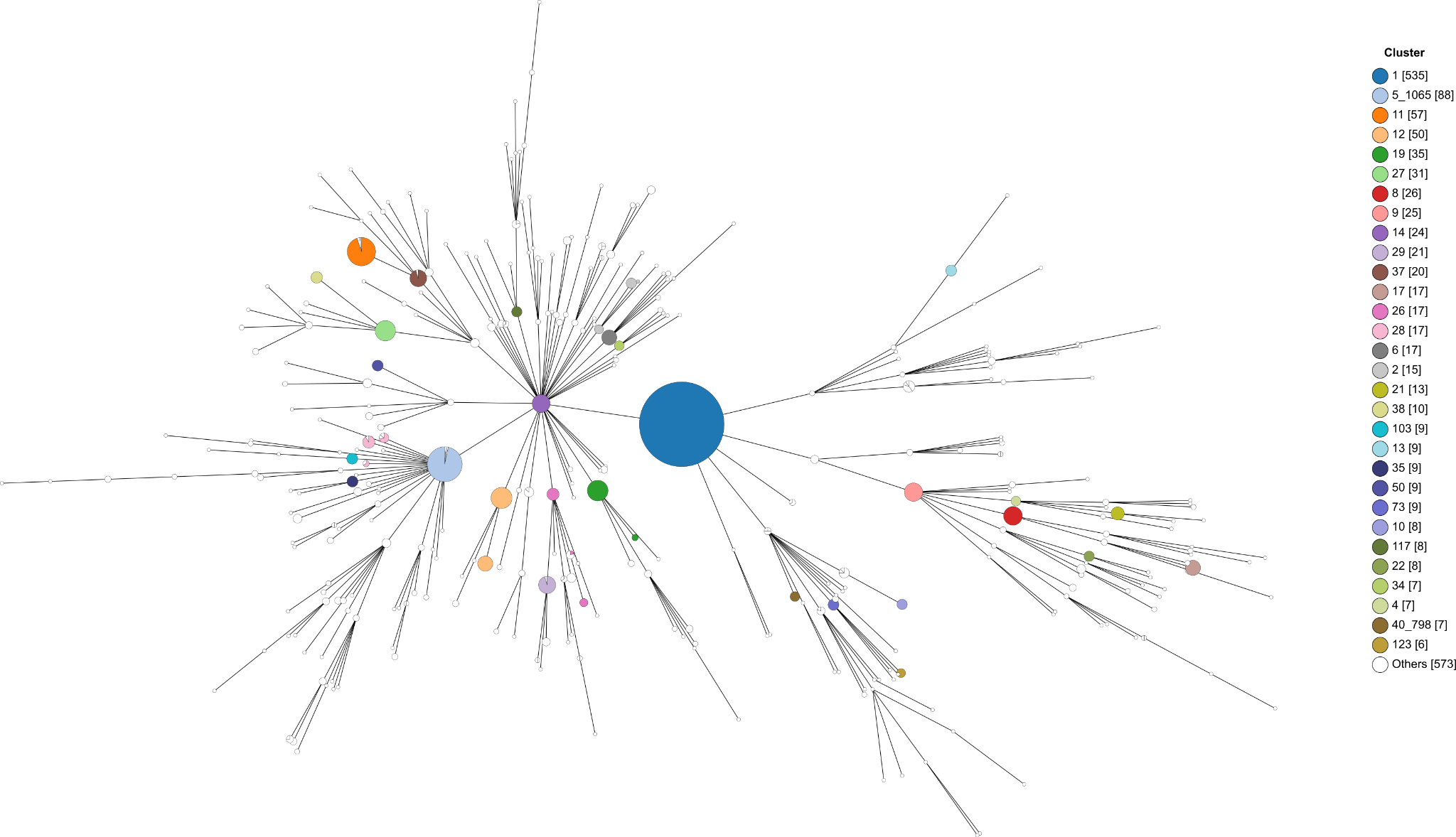


**Fig. S2.1.** cgMLST ReporTree clustering with MSTreeV2 versus PopPUNK cluster identification. The minimum-spanning tree was obtained with ReporTree using the MSTreeV2 method for the *E. coli* dataset. Branches collapsed at 724 allele differences and nodes colored according to the clusters determined by PopPUNK.

Interpretation:

This tree shows a high congruence between the two methods at this resolution level, which is supported by an Adjusted Rand coefficient of 0.995. Indeed, there is a high concordance between the clusters determined by PopPUNK and those determined by ReporTree in the stability region covering this resolution level (ranging between 590 and 730 allele differences with MSTreeV2), with a peak of congruence at 724 allele differences (Adjusted Rand = 0.995), thus showing the applicability of ReporTree for studies on population structure.


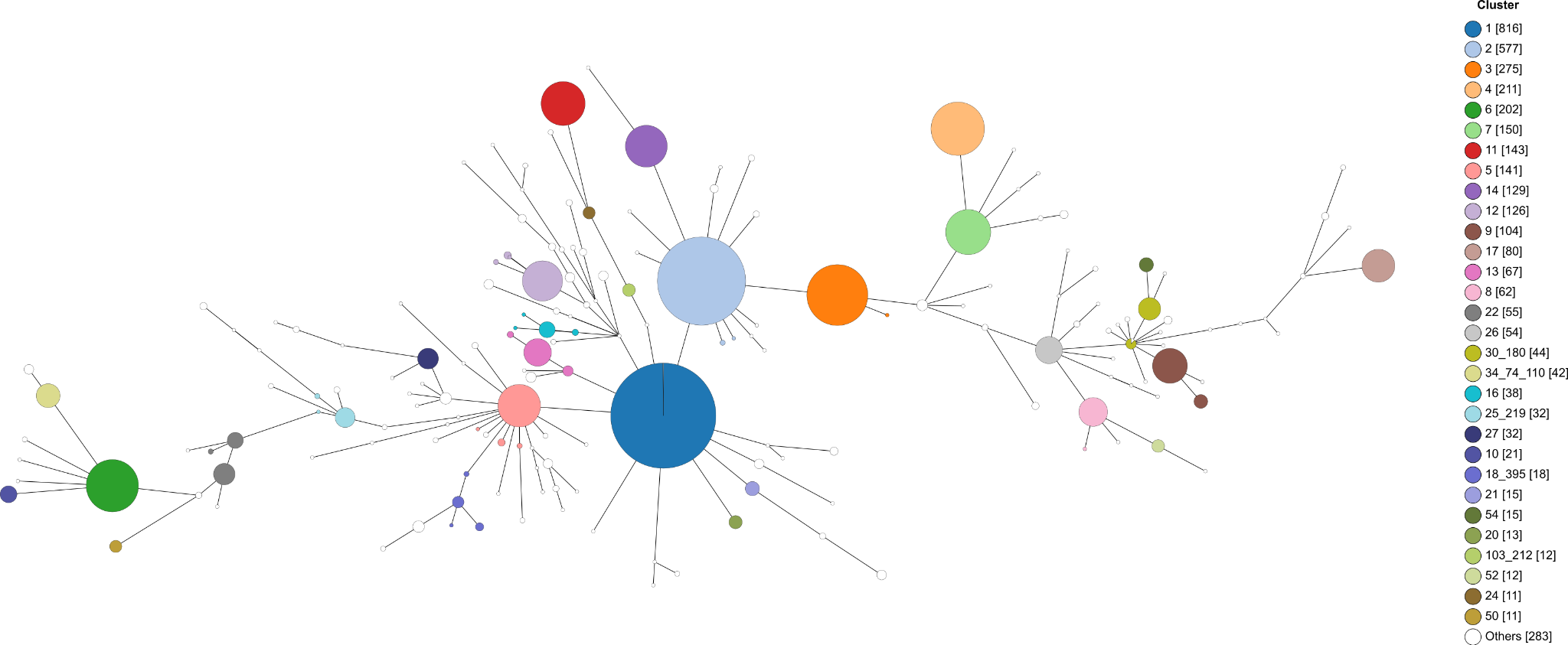


**Fig. S2.2.** cgMLST ReporTree clustering with MSTree versus PopPUNK cluster identification. The minimum-spanning tree was obtained with ReporTree using the MSTree method for the *N. gonorrhoeae* dataset. Branches are collapsed at 79 allele differences (the higher level of stability used to define genogroups in ([Pinto et al. 2021](https://www.microbiologyresearch.org/content/journal/mgen/10.1099/mgen.0.000481))) and nodes colored according to the clusters determined by PopPUNK.

Interpretation:

This tree shows a high congruence between the two methods at this resolution level, which is supported by an Adjusted Rand coefficient of 0.993. Indeed, there is a high concordance between the clusters determined by PopPUNK and those determined by ReporTree in the stability region that ranges between 79 and 200 allele differences, with a peak of congruence at 102 allele differences (Adjusted Rand = 0.996).

**Table S2.1:** Running times of each of the clustering methods used in Exercise 2.

|  | ***E. coli*** | ***N. gonorrhoeae*** |
| --- | --- | --- |
| **ReporTree (MSTreeV2)** | 1min 34s | - |
| **ReporTree (MSTree)** | - | 1min 12s |
| **ReporTree**  **(HC)** | 1min 01s | 36s |
| **PopPUNK *** | 40min 02s | 39min 42s |

**PopPUNK was run with a single thread. However, it can be parallelized taking significantly less time. Of note, the tools started from a different input (assemblies in PopPUNK and cgMLST matrix in ReporTree). Therefore, the running times might not be directly comparable, even though they reflect the clustering module of both tools.*

**Exercise 3**

Goal: Assess how core SNP-based ReporTree clustering (stability regions) compares with traditional lineage classification and also Bayesian analysis of Population Structure (BAPS)

Datasets: *M. tuberculosis* [https://zenodo.org/record/7772652](https://zenodo.org/record/7772652#.ZCGaYcrMJPY)

Input: core SNP alignment (MTb_original_align_profile.fasta)

Software settings:

*FastBAPS (*[*https://github.com/gtonkinhill/fastbaps*](https://github.com/gtonkinhill/fastbaps)*)*

version 1.0.8 (default settings)

*ReporTree (*[*https://github.com/insapathogenomics/ReporTree*](https://github.com/insapathogenomics/ReporTree)*)*

version 2.0.0 (default settings with the exception of “--site-inclusion 0.95”) using the following clustering methods:

i) hierarchical clustering (single-linkage)

ii) MSTreeV2 (GrapeTree)

Technical notes:

- All runs were performed in a laptop [Intel Core i7(R)] with 16 GB of RAM using a single thread.
- The running times only cover the ReporTree step under comparison in this exercise, i.e. the clustering.
- Adjusted Rand calculation was performed using comparing_partitions_v2.py (default settings): <https://github.com/insapathogenomics/ComparingPartitions>
- Adjusted Rand coefficient indicates the concordance of two typing methods (it varies between 0 and 1, 1 representing full concordance)

Results:


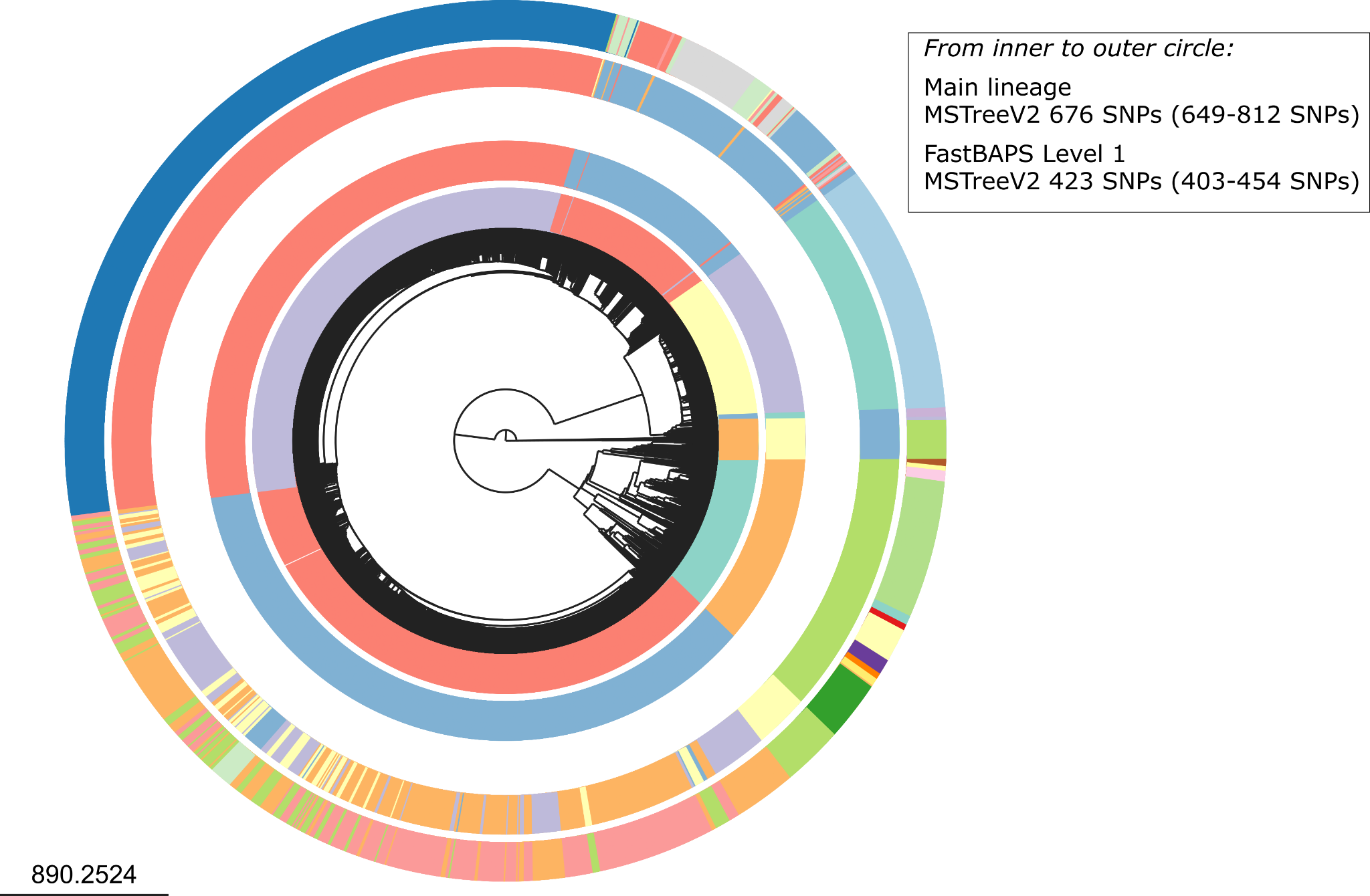


**Fig. S3.1.** core SNP-based ReporTree clustering versus lineage classification and BAPS. The figure depicts a Microreact visualization of the HC dendrogram obtained with ReporTree for the *M. tuberculosis* dataset colored according to the methods under comparison. From inner to outer circles, leaves represent traditional MTb lineage classification (determined using Tb-profiler [[Phelan et al. 2019](https://genomemedicine.biomedcentral.com/articles/10.1186/s13073-019-0650-x)]), MSTreeV2 clustering at 676 SNPs, FastBAPS level 1 clustering and MSTreeV2 clustering at 423 SNPs. Values between parentheses represent the Comparing Partition stability regions at which the points of highest congruence (676 and 423 SNPs) fall.

Interpretation:

This exercise shows that HC and MSTreeV2 provide genetic clusters that correlate well with the population structure of the dataset inferred either by the traditional MTb lineage classification or by FastBAPS. Indeed, MTb traditional lineages were found to be highly congruent with the clustering obtained at 676 SNPs, and FastBAPS level 1 with the clustering obtained at 423 SNPs, using MSTreeV2 method (applied here due to its suitability to handle core SNP alignments with missing data - “--site-inclusion 0.95”). Remarkably, these two thresholds of congruence (identified as the cutoff yielding the highest Adjusted Rand coefficient: 1 for traditional lineage classification and 0.92 for FastBAPS level 1) fall within stability regions as assessed by ReporTree’s Comparing Partitions functionality, thus supporting its utility to characterize bacterial population structure also using core-SNPs alignments as input.

*Running times:* ReporTree clustering was obtained for all possible thresholds in 1h11min using MSTreeV2 algorithm. FastBAPS clustering was obtained for two low resolution levels in 2min 25s.

**Exercise 4**

Goal: Assess ReporTree performance and usefulness to obtain and report clusters from a massive Newick tree (>6 million sequences)

Datasets: SARS-CoV-2 (Taxonium [<https://cov2tree.org/>] retrieved from <http://hgdownload.soe.ucsc.edu/goldenPath/wuhCor1/UShER_SARS-CoV-2//> on April 24^th^, 2023)

Input: Newick tree and metadata table (>6 million sequences)

Software settings:

*ReporTree (*[*https://github.com/insapathogenomics/ReporTree*](https://github.com/insapathogenomics/ReporTree)*)*

version 2.0.0 using the following clustering methods:

i) TreeCluster (avg-clade)

Technical notes:

- All runs were performed in a laptop [Intel Core i5(R)] with 16 GB of RAM using a single thread.
- The Newick tree retrieved from Taxonium had to be converted into a Newick format compatible with ReporTree. To this end, we used the script *newick4reportree.py* available at [*https://github.com/insapathogenomics/ReporTree/tree/main/scripts*](https://github.com/insapathogenomics/ReporTree/tree/main/scripts)

Results and Interpretation:

ReporTree can dive into a massive tree (and respective metadata) to extract surveillance-oriented reports and get insight on the pathogen genetic diversity. Below, we present examples on how ReporTree can answer to different questions of interest for surveillance:

1. **Zoom-in the phylogenetic branch of an emergent Pango lineage of interest** (e.g., XBB.1.16.1) **to generate surveillance report and get insight on clusters of very closely related sequences** (e.g., avg-clade distance of 2 SNPs)

*Command line:*

python reportree.py -m input/metadata.nwk -t input/tree.nwk --columns_summary_report pango_lineage_usher,country,n_country,first_seq_date,last_seq_date,timespan_days --metadata2report pango_lineage_usher,country,iso_week --method-threshold avg_clade-2 --subset -f "pango_lineage_usher == XBB.1.16.1" --count-matrix pango_lineage_usher,country:iso_week -out output/SARS-CoV-2_XBB.1.16.1

*Reports:*

**Table S4.1.1:** Lineage summary.


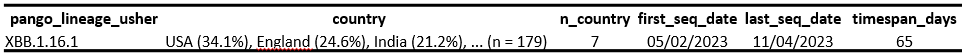


**Table S4.1.2:** Clusters of very closely related XBB.1.16.1 sequences.


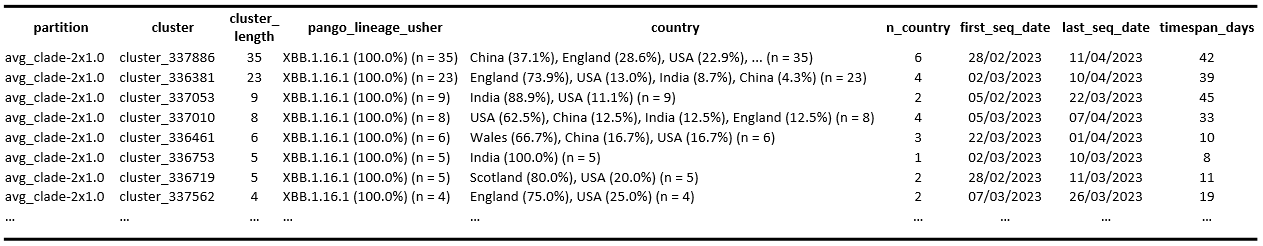


*The full table can be found at* [*https://github.com/insapathogenomics/ReporTree/blob/main/examples/SARS-CoV-2_lineage/output/SARS-CoV-2_XBB.1.16.1_partitions_summary.tsv*](https://github.com/insapathogenomics/ReporTree/blob/main/examples/SARS-CoV-2_lineage/output/SARS-CoV-2_XBB.1.16.1_partitions_summary.tsv)

**
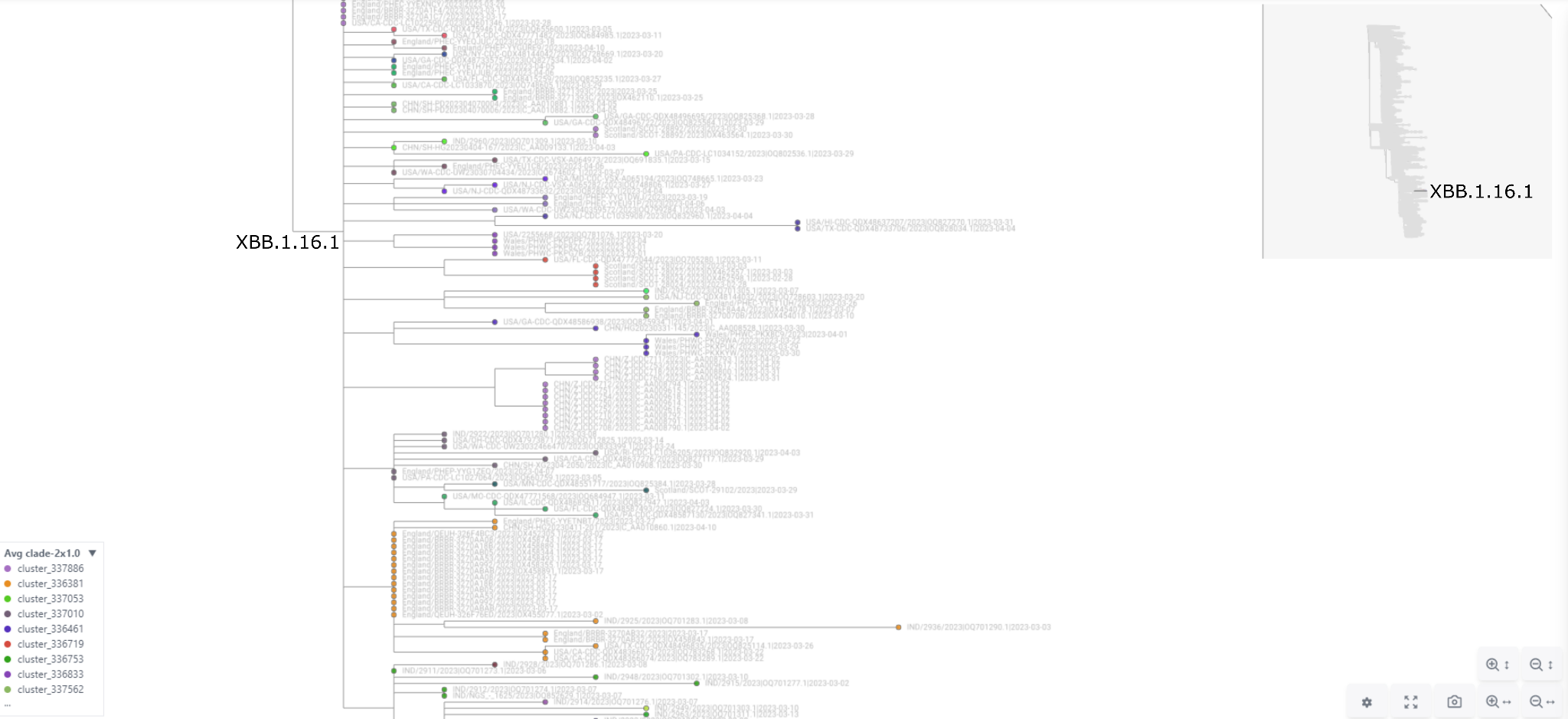
**

**Fig. S4.1** Zoom-in on the phylogenetic branch of a lineage of interest (e.g., XBB.1.16.1) from a >6 million sequences Taxonium tree colored according to the ReporTree clusters of closely related sequences (avg-clade 2).

1. **Snapshot of recent sequences** (e.g., from 2023-03-01 until the moment of data download - 2023-04-24) **to assess their geographical distribution, report their lineages and extract clusters of very closely related recent sequences** (e.g., avg-clade distance of 2 SNPs)

*Command:*

python reportree.py -m input/metadata.nwk -t input/tree.nwk --columns_summary_report pango_lineage_usher,country,n_country,first_seq_date,last_seq_date,timespan_days --metadata2report pango_lineage_usher,country,iso_week --method-threshold avg_clade-2 --subset -f "date >= 2023-03-01" --count-matrix pango_lineage_usher,country:iso_week -out output/SARS-CoV-2_last2months

*Reports:*

**Table S4.2.1:** Geographical (country) distribution of recent sequences.


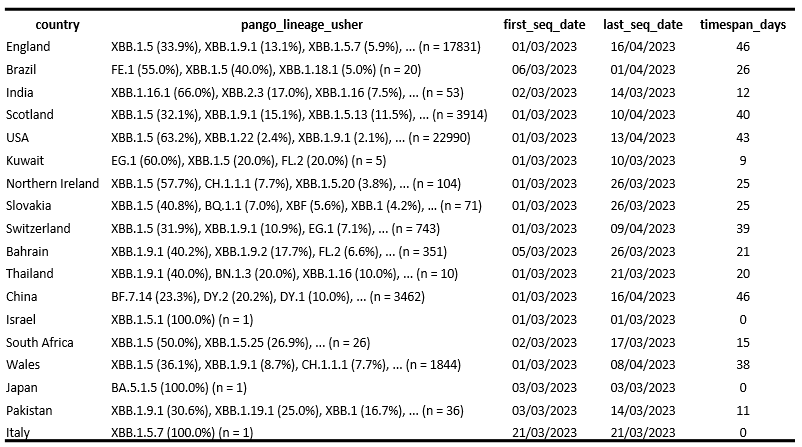


**Table S4.2.2:** Lineage distribution of recent sequences.


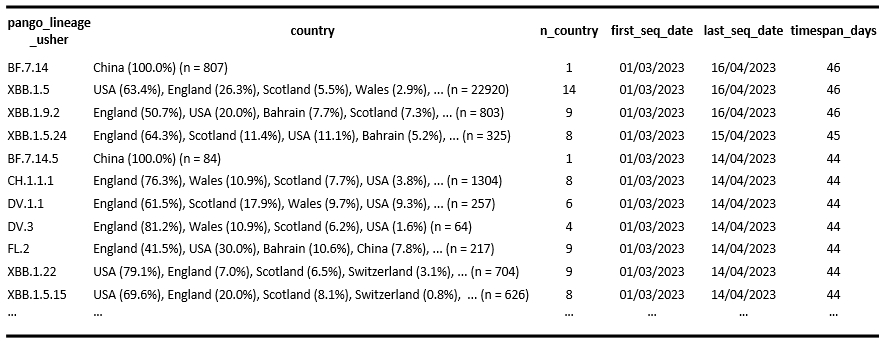


*The full table can be found at* [*https://github.com/insapathogenomics/ReporTree/blob/main/examples/SARS-CoV-2_recent_samples/output/SARS-CoV-2_last2months_pango_lineage_usher_summary.tsv*](https://github.com/insapathogenomics/ReporTree/blob/main/examples/SARS-CoV-2_recent_samples/output/SARS-CoV-2_last2months_pango_lineage_usher_summary.tsv)

**Table S4.2.3:** Clusters of closely related sequences with lineage and geotemporal characterization.


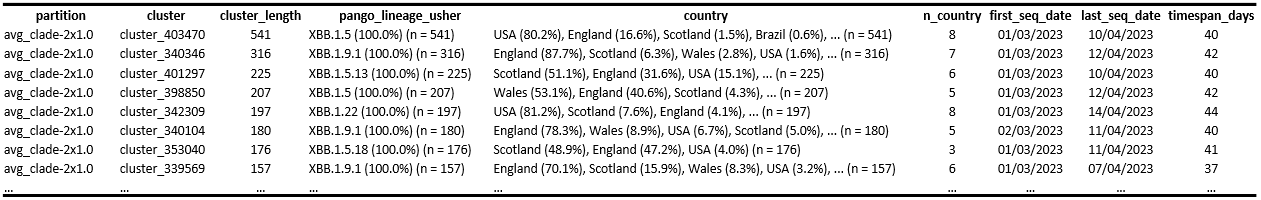


*The full table can be found at* [*https://github.com/insapathogenomics/ReporTree/blob/main/examples/SARS-CoV-2_recent_samples/output/SARS-CoV-2_last2months_partitions_summary.tsv*](https://github.com/insapathogenomics/ReporTree/blob/main/examples/SARS-CoV-2_recent_samples/output/SARS-CoV-2_last2months_partitions_summary.tsv)
